# Feasibility and effectiveness of a digital voice assistant for improving anti-osteoporosis medication adherence, and osteoporosis knowledge and attitudes, in postmenopausal women with osteoporosis: A 12-month randomised controlled trial

**DOI:** 10.1007/s11657-025-01529-0

**Published:** 2025-04-09

**Authors:** Melkamu Tamir Hunegnaw, Jakub Mesinovic, Paul Jansons, Elena S. George, Belinda De Ross, Nicole Kiss, Peter R. Ebeling, Robin M. Daly, Eugene Gvozdenko, David Scott

**Affiliations:** 1 Institute for Physical Activity and Nutrition, Deakin University, Geelong, Australia; 2 Department of Medicine, School of Clinical Sciences at Monash Health, Monash University, Clayton, Australia; 3 Great Australian Pty Ltd, Keysborough, Australia; 4 University of Gondar College of Medicine and Health Sciences, Institute of Public Health, Gondar, Ethiopia

**Keywords:** Digital health, Telehealth, Medication, Adherence

## Abstract

**Summary:**

Digital voice assistants (DVAs) are feasible for delivering a digital health intervention designed to improve osteoporosis self-management in postmenopausal women with osteoporosis. However, the DVA intervention did not improve anti-osteoporosis drug adherence, nor did it enhance osteoporosis knowledge or attitudes in this population.

**Purpose:**

To determine feasibility and effectiveness of a digital voice assistant (DVA) intervention for improving anti-osteoporosis medication adherence, and osteoporosis knowledge and attitudes, in postmenopausal women with osteoporosis.

**Methods:**

This 12-month single-blinded, randomised controlled trial included 50 postmenopausal women with osteoporosis randomised to DVA (N = 25) or control (N = 25) for 6 months, followed by a 6-month follow-up period. DVA participants received an Amazon Alexa device that delivered osteoporosis education videos, medication reminders and interactive quizzes. Control participants received emails with links to osteoporosis information. Anti-osteoporosis medication possession ratio (MPR; acceptable adherence defined as ≥ 0.8) was determined using Pharmaceutical Benefits Schedule data. Osteoporosis knowledge was measured using the Osteoporosis Knowledge Assessment Tool (OKAT) and medication attitudes were measured using the Adherence Evaluation of Osteoporosis Treatment (ADEOS-12) questionnaire.

**Results:**

The mean ± SD age of participants was 64.3 ± 6.1 years and 6-month DVA intervention adherence (number of DVA sessions accessed) was 79.5% (95%CI: 73.9, 84.9). The proportion of participants with acceptable 12-month MPRs was similar between groups (control: 86.4% [95%CI: 77.0, 93.6]; DVA: 95.0% [95%CI: 88.4, 100.0], *P* = 0.34). Mean OKAT scores improved in both groups after both 6- and 12 months, but there were no significance between groups. Changes in mean ADEOS-12 scores did not differ between baseline and 6 months in DVA compared to control (0.61 [95%CI: − 0.80, 2.03]) but worsened post-intervention from 6 to 12 months (net difference: − 1.42 [95%CI: − 2.80, − 0.06]).

**Conclusions:**

This DVA-delivered intervention achieved good adherence but did not improve medication adherence, osteoporosis knowledge, or attitudes compared with control. Future studies should target populations with poor adherence to anti-osteoporosis medication.

**Supplementary Information:**

The online version contains supplementary material available at 10.1007/s11657-025-01529-0.

## Introduction

Osteoporosis leads to increased bone fragility and fracture risk, with approximately 50% of women over the age of 50 experiencing minimal trauma fragility fractures [1]. This has a negative impact on the health-related quality of life and medical costs for postmenopausal women [2, 3]. Effective and safe pharmacotherapy is required to prevent and manage osteoporosis and lower fracture risk [4]. However, more than 50% of women with osteoporosis discontinue anti-osteoporosis medications before clinical effects on bone mineral density (BMD) can be seen [5]. Poor adherence to anti-osteoporosis medication appears to be explained, at least in part, by a lack of awareness that osteoporosis is a major risk factor for fracture [6], and beliefs that osteoporosis is not a serious health condition and/or that the risks of anti-osteoporosis medications outweigh their benefits [7, 8]. Thus, improving knowledge and beliefs about osteoporosis and its outcomes, as well as? anti-osteoporosis medication benefits and side effects, may improve anti-osteoporosis medication adherence [9].

Digital health interventions can facilitate better self-management of chronic diseases and may bridge access gaps for patients with osteoporosis living in underserviced and remote areas [10]. However, digital health programs using telephone calls or videoconferencing have generally been demonstrated to be time-consuming for health professionals and may not be ideal for supporting disease self-management [11]. Self-management programs can be delivered via applications on smartphones or computers, but these require users to have a degree of technology familiarity which can be a barrier for many older patients [12]. These issues may be addressed through the use of digital voice assistants (DVAs; e.g. Amazon Alexa, Google Home) because they enable patients to engage with information on disease self-management using only natural conversation [13, 14]. To our knowledge, no studies have explored the feasibility or effectiveness of DVA interventions for improving anti-osteoporosis medication adherence in patients with osteoporosis. Therefore, this study aimed to determine the feasibility (adherence, retention and safety) and effectiveness of a DVA intervention for improving anti-osteoporosis medication adherence, and osteoporosis knowledge and attitudes, in postmenopausal women with osteoporosis.

## Methods

### Study design and population

This 12-month, two-arm, randomised controlled trial (RCT) comprised a 6-month intervention phase with a 6-month follow-up phase. The primary outcome was a comparison of 12-month anti-osteoporosis medication adherence, and secondary outcomes included 6- and 12-month changes in osteoporosis knowledge and attitudes towards osteoporosis medication. Following 6-month assessments, both the DVA and control groups entered the 6-month follow-up phase where no further intervention was provided until the final assessments at 12 months in order to determine whether any intervention-related changes were sustained post-intervention.

Participants included 50 Australian women aged 50 years and older who reported having previously experienced menopause, received a clinical diagnosis of osteoporosis, and a current prescription of an anti-osteoporosis medication. Exclusion criteria included being non-English speaking; unable to walk across a room unaided; unable to complete an unsupervised home-based exercise program; previous fracture within 3 months; cognitive, speech or hearing problems that would interfere with the ability to interact with a DVA; and lack of access to a home Wi-Fi network.

Participants were recruited via national Facebook advertisements, and posts on the Deakin University Institute for Physical Activity and Nutrition website, between May and October 2021. Interested individuals registered by completing an online form which included a Plain Language Statement study information and consent form. Potentially eligible individuals were contacted by telephone to undergo a screening interview based on the inclusion/exclusion criteria. Participants were enrolled in the study if they met the study eligibility requirements and provided informed consent via the online consent form.

This RCT adhered to CONSORT guidelines and ethics approval was obtained from the Deakin University Human Research Ethics Committee (reference number 2021–008). The trial was also registered with the Australian and New Zealand Clinical Trials Registry (ACTRN12621000147886p).

### Randomisation and blinding

Immediately following completion of baseline assessments, participants were block randomised (computer-generated 1:1 group allocation; random block sizes of 4,6 and 8), stratified by medication prescription interval (monthly or less, 6-monthly, or annually or greater), to the DVA or control group for the 6-month intervention phase. Randomisation was performed by a member of the study team who was not involved with participant recruitment or outcome assessment.

### Intervention

#### DVA group

For the DVA intervention, we utilised ‘BuddyLink’ software developed by Great Australian Pty. Ltd. BuddyLink facilitates remote delivery of self-management programs via DVAs located in patients' homes [13]. Using the clinician-facing web portal, we developed and scheduled educational and instructional content (including images, audio, and video) for broadcasting through the DVA. Participants could access this content by speaking directly with their DVA device (Amazon Alexa Echo Show 8). We used courier delivery to provide all participants with these devices that had the BuddyLink application pre-installed and printed instructions for accessing BuddyLink and other Alexa features.

Throughout each month of the 6-month intervention period, the DVA group received three 3–5-min educational videos on exercise (week 1), nutrition (week 2), and anti-osteoporosis medication (week 3). In the 4th week of each month, participants completed an interactive quiz wherein the DVA asked the participant a series of questions related to the content provided in the previous three weeks. The educational videos (Table 1) were presented by investigators with relevant expertise in anti-osteoporosis medication (PRE; endocrinologist), exercise (PJ; accredited exercise physiologist), and nutrition (NK; accredited practising dietitian). All educational videos provided generalised information except for the second medication video, which was personalised to provide specific information on the participant’s prescribed anti-osteoporosis medication. DVAs also guided participants through three 20–25-min home-based exercise sessions per week over 6 months focusing on strength, balance and impact activities. Additionally, DVAs provided medication reminders based on the date/s participants self-reported at baseline for their next scheduled medication intake, or prescription renewal, for their existing anti-osteoporosis medication.

**Table 1:** Educational video topics delivered via Amazon Alexa to the DVA group

Month	Medication	Exercise	Nutrition
1	What is osteoporosis?	Strength training	Understanding serving sizes
2	How does your medication work?	Balance training	Dairy and calcium
3	Causes and consequences of poor medication adherence	Impact training	Dairy alternatives
4	Effectiveness of anti-osteoporosis medications	Aerobic exercise: pros and cons	Protein
5	Medication side-effects and reliable information sources	Mechanisms of impact training	Vitamin D
6	Maintaining medication adherence	Maintaining exercise behaviours	Maintaining nutrition behaviours

#### Control group

During the six-month intervention period, control group participants received six emails (one per month) with links to information available on the Healthy Bones Australia website [15]. In the first month of the intervention period, all participants received a link to the “About Bones” webpage. Subsequently, links to the calcium, vitamin D, exercise, breaking a bone, and treatment options webpages were delivered in a random order to each participant.

### Measurements

The feasibility of the DVA intervention was determined by participant retention, adherence to the intervention (number of prescribed DVA sessions engaged with), and safety. Participant retention was determined as the number of participants who completed 6 and 12-month follow-up assessments with an a priori target of ≥ 80%, while acceptable adherence was defined as accessing ≥ 66% of the provided DVA content across the 6-month intervention phase. Safety was assessed by the number of participant-reported adverse events self-reported to study investigators which could be reported during DVA sessions or at any time via email or telephone call.

Pharmaceutical Benefits Schedule (PBS) data was obtained by data linkage from Services Australia to assess the anti-osteoporosis medication possession ratio (MPR), during the 12-month study period. Australia has universal health care arrangements where prescription medicines are subsidised under the PBS. When a PBS medicine is dispensed, the administering pharmacy or hospital provides Services Australia with data including the prescription dispensed, date and identity of the patient [14]. MPR was calculated as the ratio of days with filled anti-osteoporosis medication prescriptions to total days of the study period (365). Acceptable medication adherence was defined as MPR ≥ 0.8 [16].

At baseline, 6 and 12 months, participants completed online (Qualtrics) surveys assessing demographic characteristics and health status and behaviours. Attitudes towards safety and effectiveness of anti-osteoporosis medication were determined using the Adherence Evaluation of Osteoporosis Treatment (ADEOS-12) questionnaire [17] which had a maximum achievable score of 22.

The Osteoporosis Knowledge Assessment Tool (OKAT) was used to measure knowledge of bone health [18]. The OKAT includes 20 true or false questions where correctly answered questions were coded as ‘1’, and incorrect or “do not know” responses were coded as ‘0’, with total scores ranging from 1 to 20 [19]. OKAT questions can also be categorised into themes, specifically ‘Understanding osteoporosis symptoms and the risk of fracture’, ‘Knowledge of risk factors for osteoporosis’, ‘Knowledge of preventative factors such as physical activity and diet’, and ‘Therapy availability’ [20].

### Sample size

A sample size of 50 participants (25 in each arm) was selected as this would be sufficient to detect a significant difference (α = 0.05 and power > 80%) in medication adherence during the 12-month study period using Fisher’s exact test allowing for 20% loss to follow-up, with the proportion of adherent participants (defined as MPR ≥ 0.8) anticipated to be 85% for the DVA group and 45% for the control group. This sample size would also be sufficient to detect small effect sizes (0.20 with 90% power and α = 0.05 [21] for secondary outcomes including osteoporosis knowledge and attitudes towards osteoporosis medication.

### Statistical analyses

Our main analyses followed intention-to-treat principles. Box plots and Shapiro–Wilk tests were used to assess the distribution of continuous data. Non-normal data were transformed to meet normality assumptions of parametric methods. Data are presented as either mean ± SD, median (interquartile range), or frequency (%). Fisher’s exact test was used to compare between-group proportions of adherent participants. Linear mixed models were used to compare changes in OKAT and ADEOS-12 scores between groups at 6 and 12 months. Models had an unstructured covariance matrix and included time (3 levels), group (2 levels) and a group-by-time interaction term as fixed effects, and unique participant identifiers as a random intercept. Estimated marginal means were used to calculate within- and between-group differences. Per protocol analyses were also performed including only DVA participants who accessed at least 66% of the provided DVA intervention content across the 6-month intervention phase; all control group participants were included. All data were analysed using Stata SE-17 and SPSS statistical analysis software. P-values < 0.05 or 95% confidence intervals not including the null point were considered statistically significant.

## Results

**Fig. 1:**
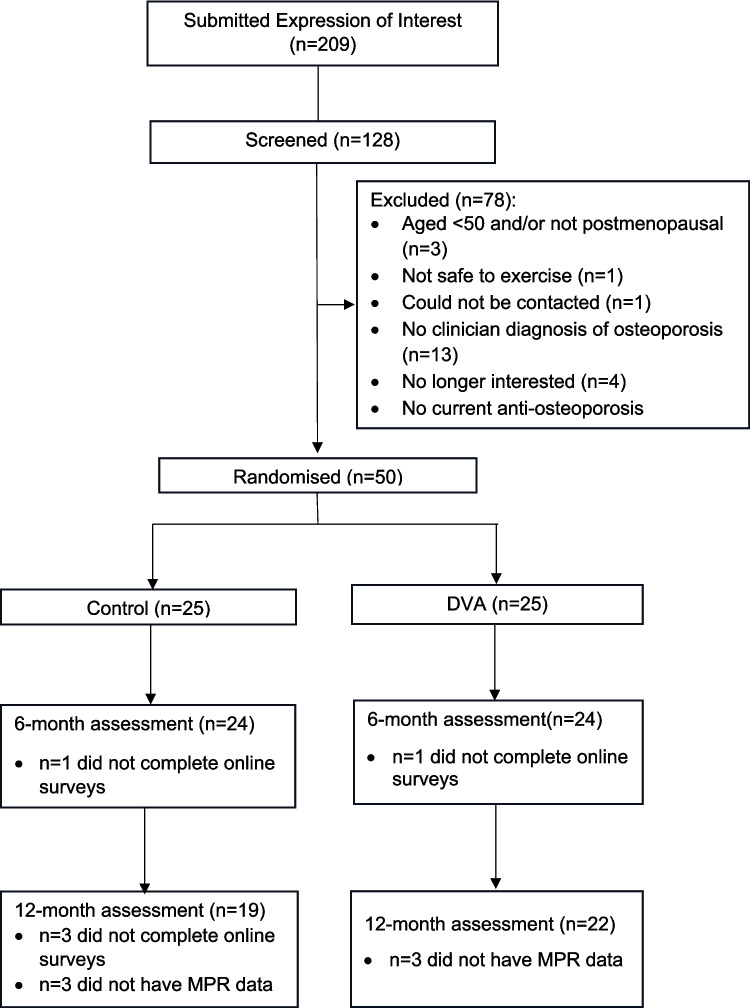
Flow diagram demonstrating the flow of participants through the study

As indicated in Fig. 1, 209 individuals completed online expressions of interest to participate in this study. After screening the initial 128 individuals, 59 (46%) eligible participants were identified; nine potential participants were added to the study waitlist, 50 were randomised to the DVA or control group and the remaining individuals were not screened further. The primary reason for ineligibility was not currently being prescribed an anti-osteoporosis medication (60.3%); four individuals chose not to participate after receiving further information about the study. There were no withdrawals during the 12-month study period although two (one DVA and one control) and three (all control) participants, respectively, did not complete online surveys at 6 and 12 months. Additionally, while all participants consented to investigators accessing their PBS records, anti-osteoporosis medication adherence data were not obtained for six participants (three DVA and three control) who could not be identified by Services Australia.

The mean ± SD age of participants included in this study was 64.3 ± 6.1 years and the mean time since menopause was 14.9 ± 0.14 years. Mean age at osteoporosis diagnosis was 59.5 ± 6.0 years, 62% of participants had been educated at a university or another higher education institution and 74% were married or had de facto marital status. Denosumab was the most reported anti-osteoporosis medication (80%) (Table 2). Intervention adherence within the DVA group during the 6-month intervention phase was 79.5% (95%CI: 73.9, 84.9) and 20 of 25 (80%) DVA participants achieved ≥ 66% adherence. Four non-serious adverse events were self-reported (all in the DVA group) including back pain, a shoulder ligament injury, a rheumatoid arthritis flare-up, and a short-term virus. These adverse events resulted in brief (1–2 weeks) reductions in engagement with the DVA exercise program, but none were deemed to be related to the exercise intervention.

**Table 2:** Baseline descriptive characteristics of DVA and control groups

Variable	DVA Group *N* = 25	Control Group *N* = 25
Age (years)	63.5 ± 6.1	65.1 ± 6.1
Age at menopause (years)	49.6 ± 10.0	49.7 ± 9.2
Age at osteoporosis diagnosis (years)	60.7 ± 5.3	58.8 ± 6.5
Highest educational status		
Secondary/high school	2 (8.00)	4 (16.0)
Technical or further educational institution	4 (16.0)	9 (36.0)
University or higher education	19 (76.0)	12 (48.0)
Marital status		
Single or widowed	5 (20.0)	2 (8.0)
Divorced or separated	4 (16.0)	2 (8.0)
Married or De facto	16 (64.0)	21 (84.0)
Employment status		
Full or part-time employed	10 (40.0)	7 (28.0)
Retired, home duties or Pension	15 (60.0)	18 (72.0)
Ant-osteoporosis medication		
Denosumab	20 (80.0)	20 (80.0)
Zoledronate, Risedronate, Alendronate	4 (16.0)	5 (20.0)
Teriparatide	1 (4.0)	-
Smoking status		
Current/Ex-smoker	8 (32.0)	7 (28.0)
Never smoker	17 (68.0)	18 (72.0)
Falls in the past 12 months		
No fall	18 (72.0)	22 (88.0)
One fall	7 (28.0)	3 (12.0)
Lifetime fracture		
Yes	19 (76.0)	21 (84.0)
No	6 (24.0)	4 (16.0)

Data are mean ± SD or frequency (%)

Mean 12-month medication adherence was 81.1% (95%CI: 74.2, 87.6) for control and 90.7% (95%CI: 86.0, 95.0) for DVA. The proportion of participants with acceptable 12-month medication adherence according to the medication possession ratio (MPR ≥ 0.8) was 86.4% (95% CI: 77.01, 93.6) and 95.0% (95% CI: 88.5, 100.0) in the control and DVA groups, respectively. There was no significant difference in mean medication adherence, or the proportion of participants with acceptable medication adherence, between control and DVA at 12 months (both *P* > 0.05).

**Fig. 2:**
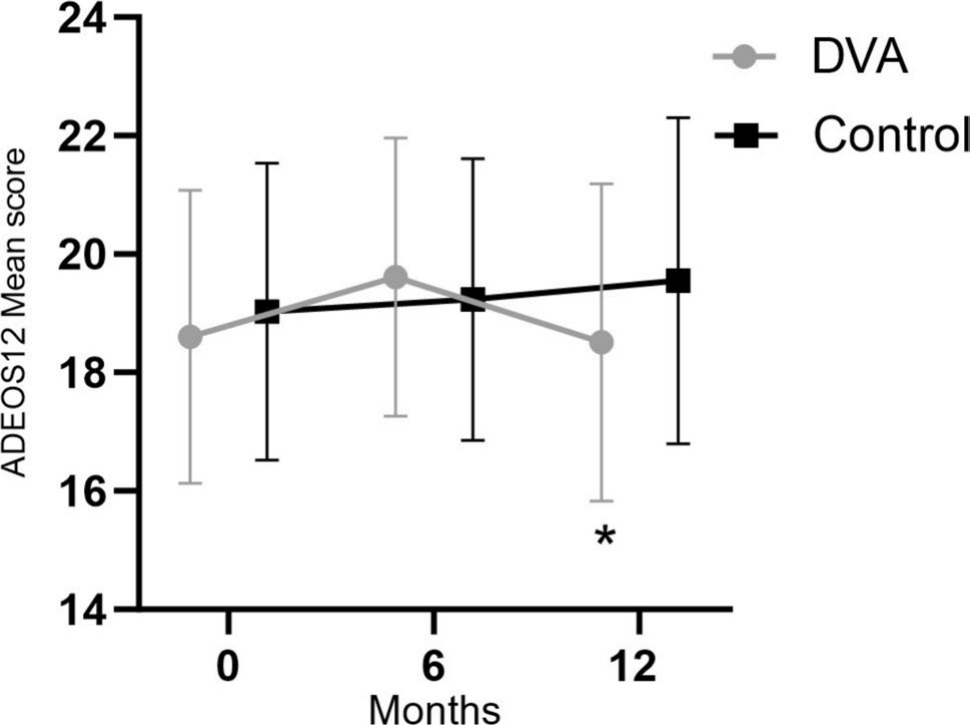
Mean ± SD Adherence Evaluation of Osteoporosis Treatment (ADEOS-12) scores for digital voice assistant and control groups at baseline, 6, and 12 months. *indicates significant (*P* < 0.05) group X time interaction between 6 and 12 months

In intention-to-treat analyses, there were no changes in mean attitudes toward osteoporosis medication (ADEOS-12 scores) for DVA or control group participants from baseline to six or 12 months in DVA compared to control (0.61[95%CI: − 0.80, 2.03]), but scores decreased significantly from six to 12 months in the DVA group compared with control (net difference: − 1.42 [95%CI: − 2.80, − 0.06], *P* = 0.04) (Fig. 2).

**Fig. 3:**
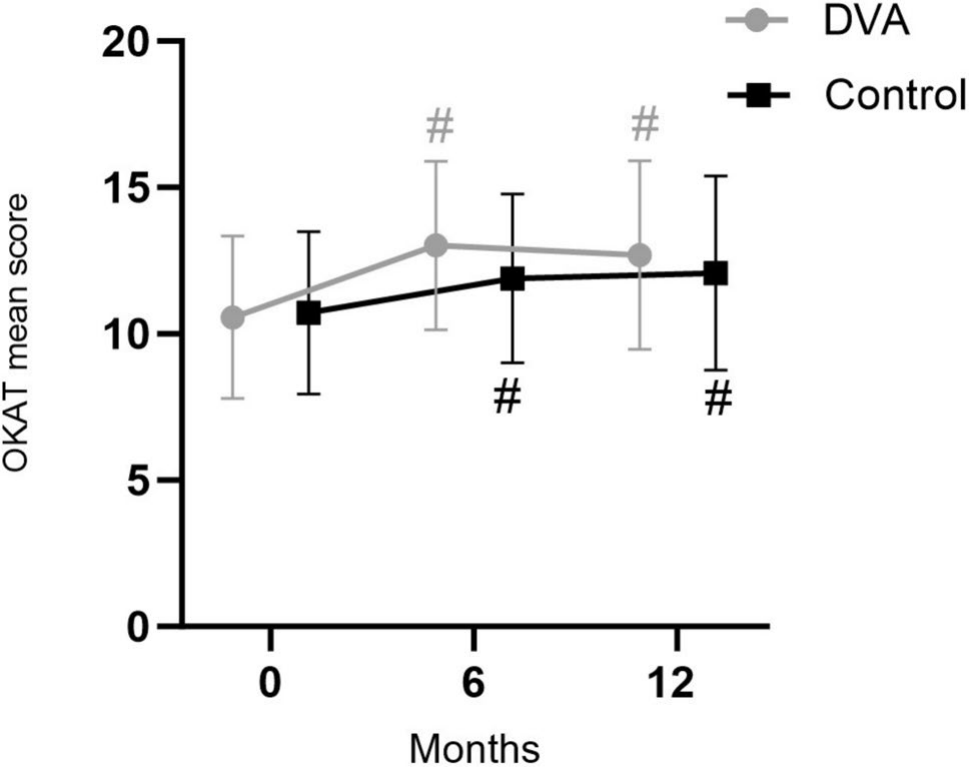
Mean ± SD Osteoporosis Knowledge Assessment Tool (OKAT) scores for digital voice assistant and control groups at baseline, 6 and 12 months. # Indicates significant (*P* < 0.05) within-group change relative to baseline

Mean OKAT scores significantly increased from baseline to six months in both the DVA group (2.46 [95%CI: 1.49, 3.44], *P* = 0.001) and the control group (1.18 [95%CI: 0.20, 2.16], *P* = 0.02) (Fig. 3). Similarly, from baseline to 12 months, mean OKAT scores increased in both DVA (2.13 [95%CI: 1.16, 3.10], *P* = 0.001) and control (1.36 [95%CI: 0.34, 2.38], *P* = 0.01). However, there were no differences between the groups for changes in OKAT scores at any time-point.

For sub-classifications of the OKAT (Table 3), the mean score for the theme “Understanding symptoms and risk of fracture of osteoporosis” significantly increased in both groups from baseline to six months. There was also a significant increase in the DVA group only from baseline to 12 months, which was higher compared with control. Mean scores for the theme “Knowledge of risk factors for osteoporosis” significantly increased in the DVA group only from baseline to six months and from baseline to 12 months. In the control group, scores significantly increased from baseline to 12 months. The mean score for the theme “Knowledge of preventive factors such as physical activity and diet relating to osteoporosis” significantly increased in the DVA group only from baseline to six months; both groups improved from baseline to 12 months. There were no significant changes in mean scores for the OKAT theme “Treatment availability of osteoporosis”, nor were there any between-group differences (Table 3).

**Table 3:** Mean baseline values and intention-to-treat analyses of between-group changes in OKAT theme score after six and twelve months in control and DVA groups

	DVA *N* = 25	Control *N* = 25	Net Difference (95% CI)	Group x Time (*P*-value)
Understanding symptoms and risk of fracture of osteoporosis				
Baseline	2.80 ± 0.96	2.56 ± 0.96		
Δ 0–6 months	**0.64 ± 0.94**	**0.43 ± 0.94**	0.21 (− 0.32,0.75)	0.43
Δ 6–12 months	0.07 ± 0.35	− 0.31 ± 1.08	0.38 (− 0.22,0.98)	0.21
Δ 0–12 months	**0.71 ± 0.91**	0.12 ± 0.60	**0.60 (0.06,1.12)**	**0.03**
Knowledge of risk factors for osteoporosis				
Baseline	3.24 ± 1.44	3.56 ± 1.44		
Δ 0–6 months	**1.07 ± 1.57**	0.47 ± 1.57	0.60 (− 0.29,1.49)	0.18
Δ 6–12 months	− 0.18 ± 1.52	0.32 ± 1.58	− 0.50 (− 1.39,0.38)	0.26
Δ 0–12 months	**0.89 ± 1.46**	**0.80 ± 1.53**	0.09 (− 0.76,0.94)	0.83
Knowledge of preventive factors such as physical activity and diet relating to osteoporosis				
Baseline	3.40 ± 1.19	3.20 ± 1.19		
Δ 0–6 months	**0.63 ± 1.08**	0.32 ± 1.09	0.31 (− 0.31,0.93)	0.32
Δ 6–12 months	− 0.08 ± 1.02	0.20 ± 1.06	− 0.28 (− 0.87,0.31)	0.35
Δ 0–12 months	**0.55 ± 1.16**	**0.52 ± 1.21**	0.03 (− 0.65,0.70)	0.93
Treatment availability of osteoporosis				
Baseline	1.12 ± 0.67	1.40 ± 0.67		
Δ 0–6 months	0.13 ± 0.65	− 0.03 ± 0.68	0.16 (− 0.23,0.55)	0.41
Δ 6–12 months	− 0.08 ± 0.48	− 0.06 ± 0.50	− 0.02 (− 0.29,0.26)	0.89
Δ 0–12 months	0.05 ± 0.62	− 0.09 ± 0.64	0.14 (− 0.22,0.50)	0.43

Within-group changes are presented as mean ± SD. Between-group differences are presented net difference between within-group changes (DVA – control) with 95% confidence intervals. Bold numbers indicate statistically significance change (*P* < 0.05)

Per-protocol analyses included DVA group participants who achieved at least 66% adherence to the intervention (DVA *n* = 17; control *n* = 22). Mean anti-osteoporosis medication adherence was 83.0% (95%CI: 69.0, 97.0) and 93.0% (95%CI: 81.0, 100.0) in the control and DVA groups, respectively. All results were unchanged except mean ADEOS-12 scores did not significantly change within the DVA group from six to 12 months and the mean score for the theme “Understanding symptoms and risk of fracture of osteoporosis” did not significantly increase in the control group from baseline to six months (Supplementary Tables 1 and 2).

## Discussion

This RCT demonstrated that a 6-month DVA self-management program did not increase the proportion of postmenopausal women achieving acceptable 12-month osteoporosis medication adherence. Osteoporosis knowledge increased in both the DVA and control group, with no significant difference between groups. Attitudes toward osteoporosis medication worsened in DVA compared with control following the 6-month intervention period. The DVA intervention was both safe and feasible with no intervention-related adverse events and demonstrating good adherence and retention (no withdrawals).

Eighty percent of participants in the DVA group completed at least 66% of the prescribed intervention with mean adherence approaching 80%. These findings indicate that this DVA intervention is feasible for self-management in patients with osteoporosis. This result cannot be compared to other DVA osteoporosis self-management interventions as it is the first of its kind that to our knowledge. However, a prospective survey study of 100 postmenopausal women with osteoporosis demonstrated that interventions using a smartphone, tablet, or computer application were feasible for improving osteoporosis self-management intervention adherence [22].

Various interventions, such as patient education, monitoring and supervision, changes in drug regimen, and interdisciplinary collaboration have been implemented to enhance anti-osteoporosis medication adherence [23]. A scoping review showed that digital health interventions including mobile apps, SMS messaging, and wearable devices can play a key role in improving medication adherence in patients with chronic diseases [24]. Studies have demonstrated that educational interventions at health centres using different interactive methods like group discussion, and question and answer sessions can improve women’s attitudes towards osteoporosis [25, 26]. However, in our study, the DVA intervention did not significantly improve medication adherence compared with control, aligning with previous telephone-based educational interventions which had no significant effect on osteoporosis medication adherence [27, 28]. This finding may be related to the apparent high levels of anti-osteoporosis medication adherence and attitudes toward osteoporosis medication observed in our cohort, with high baseline scores observed for the ADEOS-12.

Mean ADEOS-12 scores decreased significantly from six to 12 months in the DVA group compared with control, suggesting that attitudes towards osteoporosis medication worsened when the intervention ceased. While it is unclear why this finding was observed, it possibly indicates a need for ongoing education to maintain positive attitudes towards anti-osteoporosis medication. Education provided using various digital intervention methods including mobile applications, wearable devices, and web-based platforms, has been shown to improve osteoporosis knowledge among older adults [29, 30].

An interesting finding from this study was that osteoporosis knowledge significantly increased in both groups after 6 and 12 months. Consistent with this finding, a study of Korean patients aged > 50 years with osteoporosis found that educational interventions using written material significantly increased knowledge, as evidenced by increased mean OKAT scores after 3 months [29]. Conversely, another RCT revealed that mailed educational interventions had no significant effect on osteoporosis knowledge compared to the control group [31], and the 18-month Osteo-cise: Strong Bones for Life multifaceted intervention that included progressive resistance, weight-bearing impact and balance training, osteoporosis education and behavioural support, did not improve osteoporosis knowledge or beliefs [32]. In our study, improvements in participants' osteoporosis knowledge were similar between groups throughout the study. This suggests both interventions were effective in increasing osteoporosis knowledge. However, it should be noted that the DVA group had improvements in the theme "Understanding symptoms and risk of fracture of osteoporosis" from baseline to 12 months compared to the control group, indicating that the DVA intervention may have been more effective in improving osteoporosis knowledge in this area.

### Limitations

Limitations of this study include the small sample size which may have been insufficient to detect significant differences for some outcomes. The sample size was calculated based on a predicted MPR of 40 to 50% in the control group [33]. However, the observed MPR in both study groups exceeded 80%, suggesting our findings may have been influenced by a selection bias wherein women with already high adherence and/or positive attitudes to anti-osteoporosis medication were more likely to participate in a study on osteoporosis. Over 60% of our participants were educated at a university or other higher education level, and higher levels of education may be associated with better adherence to osteoporosis treatment [34], so it is possible that interventions such as this should be specifically targeted at populations with lower education status and/or knowledge or attitudes towards osteoporosis. Another factor that likely influenced the high medication adherence observed in our study was that approximately 80% of participants were receiving denosumab, a monoclonal antibody delivered via 6-monthly injections administered by a health professional. Denosumab adherence generally exceeds that of other medications with shorter dosage intervals (e.g. weekly and monthly) and which may be self-administered by the patient [35]. In this context, the difference in the proportion of participants with acceptable 12-month MPRs (control: 86.4% [95%CI: 77.0, 93.6]; DVA: 95.0% [95%CI: 88.4, 100.0], *P* = 0.34) may be meaningful, although future trials should potentially focus on patients using medications more commonly associated with poor adherence.

Given that this study included a multi-component intervention focused on exercise and nutrition in addition to medicine, participants may have prioritised lifestyle changes over medication adherence. The lack of differences in medication adherence between groups may also reflect that most participants were following 6- or 12-monthly anti-osteoporosis medication prescription schedules. As such, our findings may not be generalised to populations with poorer osteoporosis knowledge and attitudes, or those receiving medications on a more regular basis.

It is important to note that although MPR may provide a more accurate and objective measure of medication adherence than self-reported medication consumption, it assumes that the medication was taken as prescribed by the patient [36]. We did not collect any data on the proportion of the control group that accessed online educational materials provided via email links and it is possible that this intervention resulted in changes in knowledge, attitudes or behaviours which may have masked potential benefits of the DVA intervention.

## Conclusion

This 6-month DVA intervention was safe and achieved good adherence but did not improve 12-month anti-osteoporosis MPR or osteoporosis knowledge in postmenopausal women with osteoporosis. Future studies should target populations at risk for poor anti-osteoporosis medication adherence, such as those with lower education levels and/or receiving self-administered medications at regular intervals, to determine whether this intervention can effectively improve medication adherence and attitudes.

## Supplementary Information

Below is the link to the electronic supplementary material.

Supplementary Material 1(DOCX 19.6 KB)

## Data Availability

Data are accessible upon request to the corresponding author.
