# Supplementary material for: Feasibility and effectiveness of a digital voice assistant for improving anti-osteoporosis medication adherence, and osteoporosis knowledge and attitudes, in postmenopausal women with osteoporosis: A 12-month randomised controlled trial
Source: Arch Osteoporos. 2025 Apr 9;20(1):43. doi: 10.1007/s11657-025-01529-0 (PMC11982083; doi:10.1007/s11657-025-01529-0)
Supplement: Supplementary file 1 — Supplementary Material 1(DOCX 19.6 KB) [file 11657_2025_1529_MOESM1_ESM.docx]

**Supplementary Tables**

**Table S1. Mean baseline values and per-protocol analyses of between-group changes in OKAT and ADEOS12 scores after six and twelve months in control and DVA groups.**

|  | DVA  N=17 | Control  N=22 | Net Difference  (95% CI) | Group x Time  (P-value) | |
| --- | --- | --- | --- | --- | --- |
| OKAT score | | | | | |
| Baseline | 10.88±2.78 | 10.64±2.79 |  |  | |
| Δ 0- 6 months | **2.55±2.61** | **1.14±2.56** | 1.41 (0.30,3.10) | | 0.10 |
| Δ 6- 12 months | 0.16±2.35 | 0.14±2.42 | 0.02 (-1.54,1.58) | | 0.98 |
| Δ 0- 12 months | **2.71±2.17** | **1.28±2.28** | 1.43 (-0.03,2.89) | | 0.06 |
| ADEOS-12 score | | | | | |
| Baseline | 19.35±2.13 | 19.18±2.12 |  | |  |
| Δ 0- 6 months | 0.15±2.53 | 0.00±2.49 | 0.15 (1.50,1.79) | 0.86 | |
| Δ 6- 12 months | -0.68±1.81 | 0.55±1.88 | -1.23 (-2.43, -0.02) | **0.05** | |
| Δ 0- 12 months | -0.53±2.41 | 0.55±2.57 | -1.08 (-2.68, 0.53) | 0.18 | |

Within-group changes are presented as mean ± SD. Between-group differences are presented net difference between within-group changes (DVA – control) with 95% confidence intervals. Bold numbers indicate statistical significance (P<0.05).

**Table S2. Mean baseline values and per-protocol analyses of between-group changes in OKAT theme scores after six and twelve months in control and DVA groups.**

|  | DVA  N=17 | Control  N=22 | Net Difference  (95% CI) | Group x Time  (P-value) |
| --- | --- | --- | --- | --- |
| Understanding symptoms and risk of fracture of osteoporosis | | | | |
| Baseline | 2.82±1.01 | 2.59±1.00 |  |  |
| Δ 0- 6 months | **0.71±1.00** | 0.41±0.98 | 0.30 (-0.35,0.95) | 0.36 |
| Δ 6- 12 months | 0.23±1.03 | -0.29±1.06 | 0.52 (-0.16,1.21) | 0.13 |
| Δ 0- 12 months | **0.94±0.84** | 0.12±0.89 | 0.82 (0.26,1.39) | **0.01** |
| Knowledge of risk factors for osteoporosis | | | | |
| Baseline | 3.53±1.37 | 3.50±1.37 |  |  |
| Δ 0- 6 months | **0.87±1.60** | 0.46±1.57 | 0.41(-0.63,1.45) | 0.43 |
| Δ 6- 12 months | 0.08±1.61 | 0.33±1.64 | -0.25 (-1.32, 0.82) | 0.63 |
| Δ 0- 12 months | **0.94±1.39** | **0.79±1.47** | 0.15 (-0.78,1.09) | 0.74 |
| Knowledge of preventive factors such as physical activity and diet relating to osteoporosis | | | | |
| Baseline | 3.24±1.21 | 3.05±1.21 |  |  |
| Δ 0- 6 months | **0.86±1.03** | 0.36±1.07 | 0.50 (-0.17,1.17) | 0.14 |
| Δ 6- 12 months | 0.02±1.04 | 0.09±1.07 | -0.07(-0.76,0.62) | 0.83 |
| Δ 0- 12 months | **0.88±1.03** | **0.46±1.08** | 0.42 (-0.26,1.11) | 0.22 |
| Treatment availability of osteoporosis | | | | |
| Baseline | 1.29±0.60 | 1.50±0.60 |  |  |
| Δ 0- 6 months | 0.13±0.56 | -0.09±0.68 | 0.22 (-0.22,0.67) | 0.31 |
| Δ 6- 12 months | -0.19±0.45 | -0.01±0.46 | -0.18 (-0.49,0.12) | 0.22 |
| Δ 0- 12 months | -0.06±0.69 | -0.10±0.63 | 0.04 (-0.37,0.45) | 0.84 |

Within-group changes are presented as mean ± SD. Between-group differences are presented net difference between within-group changes (DVA – control) with 95% confidence intervals. Bold numbers indicate statistical significance (P<0.05).
